# Ultraviolet Light-Induced Skin Cancer and the Safety of Sunscreen Use in Pets—An Important but Under Researched Aspect of Companion Animal Health

**DOI:** 10.3390/vetsci13070605

**Published:** 2026-06-23

**Authors:** José Luis Granados-Soler, Michelle Majella Story, Rachel Allavena

**Affiliations:** School of Veterinary Science, The University of Queensland, Gatton, QLD 4343, Australia; j.granados@uq.edu.au (J.L.G.-S.); m.story@uq.edu.au (M.M.S.)

**Keywords:** UV, zinc oxide, titanium dioxide, dogs, cats

## Abstract

Ultraviolet (UV) light exposure is a recognised risk factor for dermal haemangiosarcoma (HSA) in dogs and dermal squamous cell carcinoma (SCC) in dogs and cats. These tumours cause substantial local disease and often require repeated surgery due to recurrence or de novo lesions, creating a notable welfare and financial burden. Research on preventing harmful UV exposure in pets is still in its infancy, particularly in relation to the safety of UV filters used in sunscreens. This review summarises the current evidence on UV-induced carcinogenesis, strategies to reduce UV exposure, and the safety of sunscreen ingredients in dogs and cats. UV light is strongly implicated in a range of dermatoses, from actinic keratosis to dermal HSA and SCC in dogs and cats, and the risk is likely higher in Australian pets. Indoor confinement during peak UV periods, shade, and sun-protective clothing can reduce exposure, with sunscreen an additional strategy. Sunscreen is relevant because UV-associated cancers typically develop in sparsely haired or hairless regions such as the nose and ventrum, making these areas suitable for targeted sunscreen application. Sunscreens containing non-nanoparticle zinc oxide appear safe for dogs and cats when ingestion is prevented or minimised, whereas the safety of organic UV filters remains unclear due to limited safety data in both humans and animals. Non-nanoparticle titanium dioxide is a possible alternative to zinc oxide and organic filters, but there is currently little information on its safety when ingested by dogs and cats. Overall, the available evidence supports sunscreen as a necessary component of UV-reduction strategies in pets, but substantial research is needed to determine the safety profiles of different UV filters and to establish evidence-based guidelines for their safe use.

## Simple Summary

The environmental factors that increase the risk of sun-induced skin cancers in people living in Australia also apply to their pets. Therefore, Australian pet dogs and cats are likely at increased risk of developing skin cancer. Sunscreen is an important part of sun-protection strategies for both humans and animals, but there are concerns around the risk of pets developing zinc toxicity from zinc oxide, a common sunscreen ultraviolet filter. The limited research available suggests that sunscreens containing zinc oxide are not likely to cause zinc toxicity in pets if excessive ingestion is prevented, whereas the safety of chemical ultraviolet filters is poorly understood. Titanium dioxide may be an alternative to zinc oxide and chemical filters in pet sunscreens, but further research is required. Overall, research on the safety and effectiveness of sunscreen in pets, particularly under Australian conditions, is urgently needed.

## 1. Introduction

Ultraviolet (UV) light exposure is a significant environmental risk factor for the development of skin tumours in both humans [1] and animals, including dermal haemangiosarcoma (HSA) in dogs [2,3,4] and dermal squamous cell carcinoma (SCC) in dogs [3,5] and cats [6,7]. These tumours are part of the spectrum of UV-associated solar dermatoses, alongside actinic dermatitis and actinic keratosis, a precursor of SCC [8]. Although UV-induced HSA and SCC are slow to metastasise, they cause significant local disease, recur frequently, and new lesions often arise in sun-damaged areas [5,9,10,11,12]. As a result, many affected animals undergo multiple surgical procedures over their lifetime, creating a considerable welfare and financial burden for owners. Preventing the development or progression of UV-associated skin lesions is therefore essential, particularly in animals that are predisposed to UV damage, making early identification of high-risk pets a critical component of effective prevention.

Several factors influence an animal’s risk of UV-associated skin disease, including individual susceptibility and the level of environmental exposure. Like humans, animals with sparsely haired and lightly pigmented skin are at higher risk of solar damage, including UV-associated cancers [1,2,3,6]. Many environmental factors that elevate UV light levels and skin cancer risk in humans also apply to companion animals. Australia has the highest incidence of human skin cancer in the world [13], in part due to geographic factors and lifestyle patterns that promote intense UV radiation exposure. Similarly, skin tumours represent the most common tumour type diagnosed in Australian dogs and cats, including cancers known to be associated with UV exposure [14]. Therefore, it is reasonable to expect that Australian pets with short fur, light pigmentation or sparsely haired areas that spend significant time outdoors are at heightened risk of UV-associated skin cancer, highlighting the need for effective preventative measures for these susceptible animals.

In order to prevent UV-related skin cancers, companion animals that spend a significant amount of time outside, live in high UV radiation locations (like Australia) or have reduced innate protection should be provided with physical barriers against UV light like those recommended in people, such as shade, clothing and sunglasses [10,15]. However, these measures often provide only partial protection because they do not adequately cover many of the most vulnerable areas, including regions of sparse hair or exposed skin such as the nose and pinna, where physical coverage is not feasible. As a result, sunscreen becomes an important additional method of safeguarding animals from harmful UV exposure.

Sunscreens protect against UV radiation by containing filters that reduce the amount of UV light that reaches the skin [16]. As with any product that is applied to the skin of animals, there is the potential risk of toxicity through topical absorption and oral ingestion, but research assessing the safety of UV filters in pets is lacking. In the Australian context, this is due to strict rules around the testing of sunscreen ingredients on animals, particularly in Queensland where it is effectively banned by the Animal Care and Protection Act 2001 (Qld) [17]. Whilst this provision was likely included to protect laboratory animals, it has had an unintended consequence of preventing direct, evidence-based risk–benefit analysis of sunscreen in domestic species, particularly companion animals. Additionally, there is controversy around the use of zinc oxide (ZnO) as a UV filter in pet sunscreens due to cases of toxicity in dogs following the ingestion of rash creams containing ZnO [18,19,20,21].

The goal of this review is to outline the role of UV light as a carcinogen and the methods for reducing UV exposure in relation to the development of skin cancer in dogs and cats. Literature and knowledge gaps on the safety of sunscreen ingredients in pets are summarised, with a focus on the concerns around organic and mineral UV filters. Given the significance of skin cancer in companion animals, the review calls for more research on the safety of sunscreen for use in pets, particularly regarding the potential toxicity of UV filters.

## 2. Ultraviolet Radiation: An Important Carcinogen

Sunlight, composed of various wavelengths within the electromagnetic spectrum, poses significant risks to skin health, with the effects of UV radiation being the most studied [22,23]. UV light is classified into three categories based on wavelength: UVA (315–400 nm), UVB (280–315 nm) and UVC (100–280 nm) [24]. All UVC and most UVB is absorbed by the ozone layer, and so the UV radiation that reaches the Earth’s surface primarily consists of UVA (90–95%) with some UVB (5–10%) [25]. Although UV radiation is fundamental to life on Earth and is vital for certain physiological processes in some animal species, particularly the synthesis of vitamin D, extensive evidence from animal and human studies classifies it as a potent carcinogen [1,25]. UVA penetrates into the dermis, whereas UVB only reaches as far as the epidermis due to its shorter wavelength, and both can cause DNA damage that leads to carcinogenesis [26,27]. UVA can interact with DNA directly, but it primarily causes indirect DNA damage through oxidative reactions [26,27]. While UVB is much less effective at inducing oxidative reactions than UVA, it is far more efficient at damaging DNA directly, and so it is considered the more carcinogenic of the two wavelengths [26,27]. However, recent research suggests that UVA has a more significant role in carcinogenesis than previously thought [26,27].

The effects of UV light on skin physiology, the inflammatory response to excess UV radiation known as sunburn, the protective effect of melanin, and the DNA mutations caused by UVA and UVB that contribute to skin cancer development have been extensively documented in humans [25,28,29]. However, there is comparatively little literature on the effect of UV radiation in companion animals. Nevertheless, it has been established that UV light exposure is a risk factor for dermal HSA in dogs [2,3,4] and dermal SCC in dogs [3,5] and cats [6,7]. Additionally, UV light exposure contributes to actinic keratosis, a precancerous condition characterised by inflammation, erythema and thick, scaly patches of skin [8].

In humans, the incidence of UV-associated skin cancers varies considerably across the globe due to a combination of genetic, environmental and behavioural factors. It tends to be higher in populations with less skin pigmentation, in geographical regions with high UV radiation levels, and where tanning, outdoor activities and clothing that exposes large amounts of skin are common [1]. UV radiation levels are higher when sunlight has a shorter, more direct path through the atmosphere, meaning they are increased at high altitudes, the equator, during summer, and when the sun is overhead [24,30]. Highly reflective surfaces (such as snow), thin or absent cloud cover, lower air pollution, and reduced ozone levels also increase UV radiation [24,30]. Additionally, Earth’s slightly elliptical orbit around the sun means that UV radiation is higher during summer in the southern hemisphere than it is during summer in the northern hemisphere [24,30]. Australia has many of the genetic, geographic and behavioural risk factors for UV-associated skin cancers, and so it is not surprising that Australia has the highest incidence rate of human skin cancer in the world [13].

**Figure 1:**
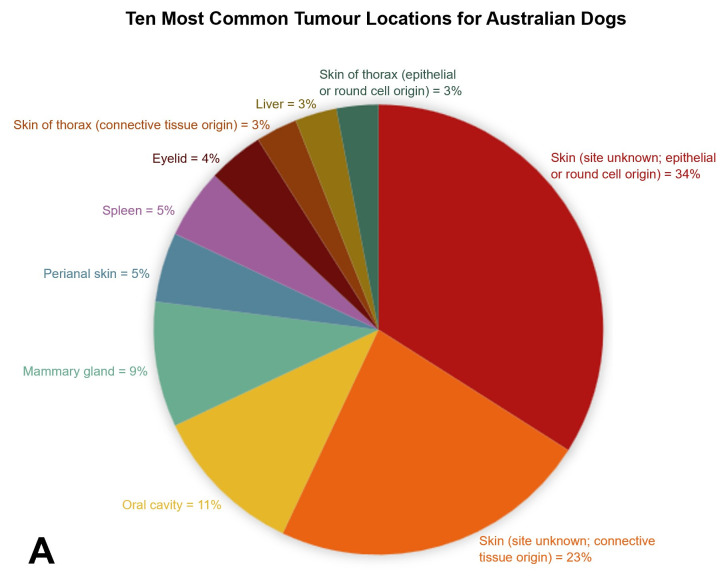

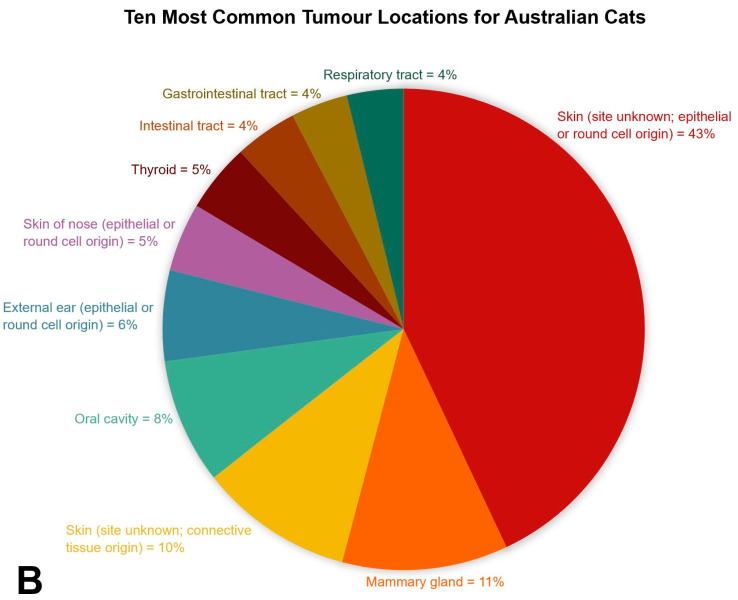
The ten most common tumour locations for Australian dogs (**A**) and cats (**B**), and the relative frequencies of each location, based on data extracted from acarcinom.org.au in May 2025 [14]. Note that skin is the most common tumour location for both species.

It can be assumed that the environmental factors that increase UV radiation exposure in humans also apply to animals. So theoretically Australian pets are at increased risk of UV-associated skin cancers. Indeed, SCC has been found to be the most common tumour type diagnosed in cats in several locations with high UV radiation, including Brazil [7], Mexico [31], South Africa [32] and central Italy [33]. In addition, HSA was the most common cutaneous tumour in a study of dogs in Grenada, West Indies, another country with high UV levels [34]. Recent large-scale studies of the prevalence of skin cancers in Australian pets are lacking, but research from the 1980s found a greater prevalence of cutaneous SCC and haemangiomas/haemangiosarcomas in dogs from North Queensland, Brisbane and Sydney compared to dogs from the United States and the United Kingdom [35,36,37]. Moreover, recent data from the Australian Companion Animal Registry of Cancers reveals that skin tumours are the most common type of tumours in Australian dogs and cats (Figure 1) [14]. Therefore, even if only a proportion of these skin tumours can be directly attributed to UV light exposure, it still represents a significant burden of disease. Consequently, preventing pets from being exposed to harmful levels of UV radiation is an important aspect of ensuring their health and welfare, particularly in locations with high UV levels like Australia.

## 3. Challenges in Providing UV Protection to Pets

Areas of lightly or unpigmented skin are most at risk of developing actinic keratosis and skin cancer, particularly in regions of reduced or absent hair coverage [2,3,6]. Consequently, there is an opportunity for selective breeding to reduce the risk of UV-related skin conditions by selecting for animals with more pigmented skin in high-exposure areas. Some research has explored the genetic background of phenotypic traits related to sun sensitivity and protection across different species [38,39,40], setting the ground for advances in this area. However, it would take many years for selective breeding to influence population and individual risk; thus, alternative methods of UV protection are required. In addition, selective breeding for dark colour may be rejected by pet owners and breeders, for whom appearance is often an important consideration.

In companion animals such as dogs and cats, exposure to UV light depends greatly on their lifestyle, whether they are kept indoors, outdoors or a combination of both. It is commonly recommended that high-risk pets be kept away from the sun during peak UV times [10,15]. However, many pets love sunbathing and will choose to lie in the sun even if provided with shade or shelter. Therefore, ensuring avoidance of the sun during peak UV levels generally requires confining the pet inside. In some regions, UV radiation levels are high for most of the day during summer, requiring the pet to be kept inside for many hours at a time, which is not appropriate for all pets and owners. Further, UV light penetrates through glass, so animals can be exposed when inside unless windows are fitted with a UV filter or an opaque covering [10,15]. Similarly, providing shade will not protect pets who choose to sunbathe or where UV exposure occurs due to reflective surfaces.

Sun-protective clothing and accessories are an option when confinement inside and shade provision are not sufficient for preventing UV exposure, but they are not appropriate in all situations. For example, clothing may not be suitable during hot weather, which is often the time when sun protection is most required, due to the risk of overheating. Furthermore, clothing and accessories, such as hats and sunglasses, will not be tolerated by all pets, plus they typically do not cover all commonly affected areas, like the nose and pinna. Therefore, while confinement inside, shade and protective clothing and accessories are important strategies for preventing excessive UV exposure in pets, they are unlikely to be sufficient, so UV mitigation methods such as sunscreen are required.

Sunscreens protect against UV radiation by containing filters that absorb, scatter or reflect UV light, thereby reducing the amount that reaches the skin [16]. They are a practical option for protecting skin that is difficult to cover by other means, such as the face. However, advising on the use of sunscreen is challenging in veterinary medicine due to the lack of information about the effectiveness and safety of sunscreen for pets. Currently, there is just one study on the effectiveness of sunscreen use in dogs, which found that sunscreen did reduce sunburn and pigmentation in crossbred Mexican hairless dogs [41]. However, due to the study’s short duration, it did not assess if sunscreen prevented actinic keratosis and skin cancer. Additionally, there is debate about the safety of ZnO as a UV filter, due to zinc toxicity in dogs ingesting ZnO rash creams [18,19,20,21]. Lastly, there are recent concerns about potential negative effects of certain UV chemical filters on environmental and human health [42].

## 4. Safety of UV Filters Used in Sunscreens

Two types of UV filters are used in sunscreens—inorganic (physical/mineral) filters, which work by absorbing, reflecting and scattering UV light, and organic (chemical) filters, which absorb UV light [16].

There is increasing concern about the safety of certain UV filters [42]. The United States Food & Drug Administration (FDA) classifies UV filters into three categories: generally recognised as safe and effective (GRASE) (category I), not GRASE due to safety issues (category II), and not GRASE because additional safety data are needed (category III) [43]. The FDA has recently proposed that only two inorganic filters, ZnO and titanium dioxide (TiO_2_), can be classified as category I and that all the currently available organic filters be classified as category II or III [43].

### 4.1. Organic (Chemical) UV Filters

Multiple concerns about organic UV filters have developed over the years, including evidence of systemic absorption and questions about their effectiveness [42]. The safety concerns in humans and animals for UV filters classified as category II and III by the FDA are summarised in Table 1. There is a lack of direct research on companion animals regarding the safety and efficacy of organic UV filters. Until this research is conducted, data can only be extrapolated from human and laboratory animal studies.

Notably, all these chemicals, including both category II filters, are currently approved for use in Australian sunscreens. For some of the category III filters, the safety concerns appear minor. However, comprehensive research into the safety of these chemicals has not been performed, in medical or veterinary contexts, thus the current lack of data does not guarantee safety. In addition, degradation after exposure to UV light has been demonstrated for several of the filters [42]. While they are usually combined with other ingredients to enhance their stability, questions remain about how effective these filters actually are [42]. It is therefore vital that high quality research assessing the safety and effectiveness of organic UV filters is performed as soon as possible. However, the current ban preventing sunscreen research of any kind in animal models in Queensland, which has very high rates of UV-associated skin cancer [77], is a significant roadblock to urgently required research. This ban is likely to prevent critical studies on the safety and efficacy of various sunscreen ingredients for human and veterinary use.

The fact that the FDA believes there is insufficient evidence to conclude that any of the current organic filters are safe for humans makes it difficult to recommend these filters for pets. Dogs and cats have a much greater risk of toxicity through ingestion, plus an increased risk of transdermal absorption because they have a thinner epidermis than humans. Therefore, it would seem that sunscreens that contain inorganic filters may be the best option for pets, given they are category I. However, they too are not without potential problems.

### 4.2. Inorganic (Physical/Mineral) UV Filters

#### 4.2.1. Zinc Oxide

ZnO provides UV protection across all UVB and the vast majority of UVA wavelengths, making it a popular broad-spectrum inorganic UV filter [42].

Zinc is an essential mineral and the second-most abundant biometal after iron [78]. It is found throughout the body and plays a vital role in numerous biological functions, including cell replication, metabolism of proteins and carbohydrates, immune response, maintenance of cell membrane structure, and stabilisation of nucleic acids [78]. About 85–90% of the body’s zinc is stored in muscles, bones and teeth, with additional amounts in the liver, skin and hair [78]. Zinc levels are carefully regulated through absorption, distribution and excretion [78].

Zinc toxicity in pets primarily arises from the ingestion of metallic objects [79]. Dogs’ curiosity and habit of ingesting foreign objects make them particularly vulnerable to zinc toxicity and, consequently, they are the most affected domestic species. Metallic zinc objects that are ingested are gradually dissolved in gastric acid, causing release of soluble zinc salts which are then absorbed into the circulation [80,81]. Presenting clinical signs include vomiting, diarrhoea, lethargy, inappetence and pigmenturia [79,82,83,84]. Clinical pathology changes include regenerative anaemia, neutrophilic leucocytosis, haemoglobinemia, bilirubinaemia, haemoglobinuria and proteinuria, and elevated amylase, lipase, blood urea nitrogen, creatinine and hepatic enzymes [79,82,83,84]. The anaemia is haemolytic with Heinz bodies and/or spherocytes [79,82,83,84]. Removal of the ingested zinc object usually rapidly decreases zinc levels [85], and survival rates are reported to be above 80% [82,83].

Topical ZnO products such as rash creams are common in households and present a risk of zinc toxicity. However, ZnO is highly irritating to the stomach, therefore ingestion usually leads to profuse vomiting, which prevents toxicosis [86]. Nevertheless, ongoing ingestion might result in toxicity and four cases of this in dogs have been reported in the literature [18,19,20,21]. While zinc toxicosis has been reported in a cat after ingestion of a metal screw [87], there are currently no reports of zinc toxicity in this species after ingestion of ZnO creams.

##### Zinc Oxide Cream Toxicity in Dogs

All four cases of zinc toxicosis in dogs due to ingestion of topical ZnO demonstrated clinical signs, clinical pathology abnormalities and outcomes similar to those seen in zinc toxicity secondary to ingestion of metallic objects. The first of these cases involved a 24.6 kg, 6-year-old male neutered Shetland Sheepdog, who was treated with ZnO ointment for four days to prevent dermatitis secondary to loose stools following surgery for a rectal mass [19]. The dog was observed to continually lick off the ointment. After three days of treatment with the ZnO ointment, the dog vomited and became inappetant. The next day, he was pyrexic, dehydrated and oliguric. Serum biochemistry showed increased alkaline phosphatase, blood urea nitrogen, creatinine, amylase, lipase and phosphate, and decreased blood glucose and sodium. Haematocrit was normal at this time. On urinalysis there was isosthenuria, proteinuria, haematuria, bilirubinuria and numerous casts. Plasma and urine zinc were both elevated. Plasma zinc was just over 15 µg/mL and urine zinc just under 20 µg/mL, whereas they were less than 10 µg/mL and less than 5 µg/mL, respectively, in samples acquired from healthy dogs and dogs with renal or hepatic failure. The dog was given intravenous fluids and one dose of furosemide, and the ampicillin that was being administrated post-surgery was changed from oral to intravenous. Fluid therapy was tapered off after 36 h as the dog had improved clinically and blood urea nitrogen, creatinine and phosphate had normalised. Two days later, the pyrexia had worsened and the dog was depressed and showing signs of abdominal pain. Haematology demonstrated poorly regenerative anaemia and neutrophilia with a left shift. Treatment with intravenous fluids, gentamicin and prednisolone was initiated, and a blood transfusion was also given. The dog had improved within three days and was discharged after six days. The haematocrit was normal at a recheck two weeks after discharge.

The second case was a 3.5 kg, 3-year-old female entire Pomeranian cross, whose owner had treated flea allergy dermatitis of the dorsal lumbar region with a lotion containing ZnO and zinc carbonate for two months, followed by a cream containing ZnO for one month [20]. The owner reported that the dog had continued to chew the affected skin despite the topical treatment. The dog was presented for veterinary treatment due to two days of vomiting. The owner had also found a large amount of blood-stained fluid. On initial presentation, the dog was pyrexic and moderately dehydrated and was given supportive treatment before being sent home. At re-examination 18 h later, she was depressed, lethargic and icteric, and hepatomegaly was suspected on abdominal palpation. There had been no further vomiting, but she had been passing blood-stained fluid from the vulva. Abdominal radiographs did not show any abnormalities. On haematology, there was a regenerative anaemia with spherocytosis and Heinz bodies, as well as neutrophilia with a left shift and monocytosis. Biochemistry results revealed elevated amylase, lipase, alkaline phosphatase, alanine aminotransferase, aspartate aminotransferase and creatinine kinase, plus mild hypokalaemia, hyperchloraemia and hypercalcaemia. The dog was given a blood transfusion, intravenous fluids, amoxycillin and prednisolone. She improved clinically within 48 h and was discharged after 72 h. The plasma zinc result was received after the dog was discharged and showed marked elevation at 270 µmol/L (reference range: 7.6–22.9 µmol/L). A blood test taken at eight days post initial presentation showed that plasma zinc was still elevated but markedly improved and that the anaemia, inflammatory leukogram and biochemical abnormalities had partially resolved.

The next case involved a 5.6 kg, 6-year-old female neutered Poodle cross who had been treated with a ZnO containing rash cream for seven days due to diarrhoea scalding [21]. The owners reported that the dog was often seen with the cream on her nose. She was referred to an emergency clinic after presenting to her regular clinic for inappetence, collapse and passing red urine. On presentation to the emergency clinic, she was dull, tetraparetic, tachycardic and tachypnoeic. Haematology demonstrated a regenerative anaemia with spherocytosis and Heinz bodies, neutrophilia with a left shift, monocytosis, and lymphopaenia. Serum biochemistry revealed elevated aspartate aminotransferase, creatine kinase and blood urea nitrogen, mild hypokalaemia, and mildly decreased thyroxine. Activated partial thromboplastin time was prolonged but prothrombin time was normal. The dog received a blood transfusion, intravenous fluid, s-adenosyl methionine, cephalexin and prednisolone. She improved rapidly and was discharged after three days. The serum zinc level, which was received after discharge, was 237.1 µmol/L (reference range: 7–25 µmol/L). Haematology performed a week after discharge showed resolution of the red blood cell abnormalities.

The most recent case involved a 2.3 kg, 2-year-old female neutered Maltese terrier who was treated with a ZnO cream for seven days due to diarrhoea scalding [18]. The owners were aware that the dog was licking the treated area. The dog presented for vomiting, lethargy and brown urine. Physical examination was normal apart from tachycardia and slightly pale mucous membranes. No effusion was present on abdominal and thoracic ultrasound. Haematology demonstrated regenerative anaemia with spherocytosis and neutrophilia. Biochemistry showed increased blood urea nitrogen, aspartate transaminase, alkaline phosphatase, gamma-glutamyl transferase, amylase and lipase, plus bilirubinaemia, hyperphosphataemia, hyperglobulinaemia and hypokalaemia. Activated partial thromboplastin time was prolonged but prothrombin time was normal. Urinalysis showed proteinuria, bilirubinuria and haematuria. The dog was initially treated with intravenous fluids, pantoprazole, maropitant and doxycycline. She later received a blood transfusion due to worsening of the anaemia, plus diphenhydramine and dexamethasone due to a transfusion reaction. A whole-body radiograph did not demonstrate the presence of any metallic foreign bodies. The anaemia and clinical signs improved after the blood transfusion and the dog was discharged three days after presentation. The plasma zinc result returned after discharge and was significantly elevated at 24 ppm (normal range: 0.7–2.0 ppm). The anaemia had improved at a recheck the day after discharge.

##### Zinc Oxide Sunscreen Risk Analysis in Dogs

Concerns about zinc toxicity have resulted in many pet care information sources advising against the use of sunscreens containing ZnO. While the four previously reported cases indicate that toxicity from ingestion of a topical ZnO cream can occur, the risk from sunscreens appears low. Concentrations of ZnO in rash creams are often as high as 40% and they are applied to areas of damaged skin to treat conditions like dermatitis, providing a long-lasting wet barrier that is easy to lick off. In contrast, sunscreen formulations typically contain ZnO in concentrations up to 25% and are designed to dry quickly, making them much more difficult to ingest. As the median toxic dose is 100 mg/kg of zinc salts for dogs [78], a 10 kg dog would need to ingest about 4 g of sunscreen containing 25% ZnO. Given these products are typically applied to relatively small areas that have reduced or absent fur such as the nose and ears, it seems that much less than this would usually be applied. However, animals that require repeat applications of sunscreen over an extended period, or that require a large amount of sunscreen due to having little or no hair, could theoretically be at risk of zinc toxicosis.

In addition to concerns about the oral toxicity of zinc, its absorption through the skin is not well understood. In particular, there is concern over the transdermal absorption of ZnO nanoparticles. Metal oxide particles greater than 100 nm can lead to an undesirable visible white film on the skin and so ZnO nanoparticles (<100 nm) are often used in human sunscreens to improve aesthetics [88]. However, nanoparticle size means they may be more easily absorbed through the skin. In a study of people where sunscreen was applied twice daily to the back for five days, absorption of zinc into the blood occurred with both nanoparticle and non-nanoparticle ZnO sunscreens [89]. The women in the nanoparticle group absorbed more zinc than those in the non-nanoparticle group, although no such difference was found for men [89]. However, even for the women in the nanoparticle group, the amount of zinc absorbed from the sunscreen was tiny compared to the normal amount of zinc present in the blood [89]. A study on hairless mice also demonstrated greater zinc absorption of nanoparticle ZnO compared to non-nanoparticle ZnO, but total body zinc stayed the same, suggesting sunscreen-derived zinc was exchanged for endogenous zinc [90]. It is important to note that it would not be necessary to use nanoparticle ZnO in sunscreens that are specifically designed for animals, as the white cast caused by non-nanoparticle ZnO is unlikely to be a cosmetic concern. In fact, the visible film of the sunscreen could be helpful for owners monitoring the presence and persistence of the cream on their pets, as it allows them to visually assess if the pet likely still has protection from the sunscreen.

Another concern is that exposure of ZnO to UV light creates reactive oxygen species (ROS) that can cause cytotoxicity and genotoxicity [91]. However, it should be noted that UV light causes significant ROS production in the skin, and so the use of ZnO may result in an overall reduction in ROS damage by limiting the amount of UV light that reaches the skin.

#### 4.2.2. Titanium Dioxide

TiO_2_ provides protection across the UVB wavelengths but may only cover a portion of the UVA, depending on the form used [42]. This means it is usually necessary to combine it with other UV filters, typically organic UV filters which, as discussed above, lack robust safety or efficacy data. This contrasts with ZnO which has a broader spectrum of UV protection.

TiO_2_ is considered biologically inert, and is used in food, cosmetics and medicines [42]. Titanium alloys are highly resistant to corrosion and biocompatible and are thus commonly used for orthopaedic implants [92]. Similar to ZnO, there are questions about the dermal absorption of TiO_2_, particularly nanoparticle TiO_2_. However, a study on minipigs found that dermal penetration of nanoparticle and non-nanoparticle TiO_2_ was low [93], plus it would not be necessary to use nanoparticle TiO_2_ in sunscreens that are designed for animals. As with ZnO, TiO_2_ has been shown to produce harmful ROS when exposed to UV radiation [94] but its ability to reduce the amount of UV light that reaches the skin might result in a net reduction in ROS-induced damage.

The vast majority of studies on the effects of oral ingestion of TiO_2_ have focused on nanoparticle TiO_2_ due to safety concerns around its use as a food additive [95]. However, rats fed non-nanoparticle TiO_2_ did not show any significant toxic effects [96]. Additionally, a study of 136 human patients with titanium implants found that blood titanium levels were elevated but did not appear to cause adverse effects [92]. Therefore, TiO_2_ is a potential option for those seeking alternatives to zinc-based sunscreens, but the safety and effectiveness of TiO_2_ as a UV filter for pets should be further investigated to confirm its suitability, given its reduced blockage of the UV spectrum.

## 5. Conclusions

The literature supports the role of UV light in the development of skin cancers in pets and indicates that the risk is likely higher in Australia compared to other countries, though more comprehensive research is required. Therefore, preventing harmful UV light exposure, particularly in high-risk animals, is vital for maximising health and welfare. Sunscreen has a role as a preventative strategy; however, the lack of safety data on UV chemical filters and the need to mitigate risks from mineral filters in companion animals supports the need for more evidence-based risk–benefit research.

There is a need to disseminate information about the toxicity and risk–benefit of various UV filters to the veterinary profession and pet owners in Australia. Despite the concerns around the use of sunscreens containing ZnO, the current evidence suggests that they should generally be considered safe in dogs and cats when ingestion is prevented or minimised, and any concerns about transdermal absorption could be reduced by using non-nanoparticle ZnO. However, further research into the effectiveness of ZnO sunscreen in preventing UV-associated skin cancers in pets does need to be performed to determine efficacy and benefit. Conversely, it is difficult to unequivocally recommend the use of organic UV filters in pets unless more comprehensive safety data becomes available. TiO_2_ may be an alternative to ZnO and organic filters, but it has a more limited spectrum of UV protection and further research into its safety and effectiveness as a pet sunscreen is needed. Given these findings, there is a clear need for research to evaluate the safety and efficacy of sunscreen use in companion animals, particularly under Australian environmental conditions. However, current regulatory restrictions limit the ability to conduct the direct studies required to generate this evidence. Further epidemiological investigation of the burden of UV-associated disease in pets is urgently needed to better define the risk–benefit profile of sunscreen use. Combined with studies assessing the safety, efficacy, and optimal application of sunscreen formulations in companion animals, such research would substantially improve our understanding of UV-related disease prevention. Given the well-established role of ultraviolet radiation in the pathogenesis of skin cancer and the significant morbidity associated with these conditions, effective mitigation strategies could provide considerable population-level health benefits for pets living in regions with high UV exposure.

## Figures and Tables

**Table 1:** Safety concerns for organic UV filters classified as category II (not generally recognised as safe and effective due to safety issues) and category III (not generally recognised as safe and effective because additional safety data are needed) by the FDA.

	UV Protection	Safety Concerns
**Category II **		
PABA (Para-aminobenzoic acid)	UVB	In humans, is associated with significant rates of allergic and photoallergic contact dermatitis, causes cross-sensitisation with other common chemicals, and is absorbed through the skin [43].
Trolamine salicylate	UVB	Risk of salicylate toxicity and coagulopathy due to absorption through the skin in humans [43].
**Category III**		
Avobenzone (Butyl methoxydibenzoylmethane)	UVA	Rapidly degrades in sunlight unless stabilised [44]. Absorbed through human skin [45,46,47]. Linked to toxic effects in zebrafish [48,49].
Cinoxate (2-ethoxyethyl p-methoxycinnamate)	UVA and UVB	Reports of photoallergic contact dermatitis in people [50,51]. Obesogenic effect demonstrated in vitro [52].
Octinoxate (Octyl methoxycinnamate)	UVB	Absorbed through human skin [47,53]. Linked to endocrine disruption in rats, mice and fish [54].
Oxybenzone (Benzophenone-3)	UVA and UVB	Absorbed through human skin [46,47,53]. Conflicting results in studies of adverse health impacts in humans [55]. Established allergen in people [56]. Linked to adverse effects in aquatic species [57].
Dioxybenzone (Benzophenone-8)	UVA and UVB	Linked to toxic effects in rats, mice and zebrafish [58,59,60,61,62,63].
Ensulizole (2-phenylbenzimidazole-5-sulfonic acid)	UVB (minimal UVA)	Linked to toxic effects in molluscs [64,65], and oxidative stress and endocrine disruption in zebrafish [66,67].
Homosalate (3,3,5-trimethylcyclohexyl 2-hydroxybenzoate)	UVB	Absorbed through human skin [47]. Linked to endocrine disruption in zebrafish [48] and rats [68].
Meradimate (Menthyl anthranilate)	UVA	Reports of photoallergic contact dermatitis in people [69,70].
Octisalate (Octyl salycilate)	UVB	Absorbed through human skin [47]. Linked to endocrine disruption in zebrafish [48].
Octocrylene (Octocrilene)	UVA and UVB	Absorbed through human skin [45,46,47]. Linked to toxic effects in aquatic species [49,54,64,71].
Padimate O (2-ethylhexyl 4-dimethylaminobenzoate)	UVB	Linked to endocrine disruption in rats [68]. Possibly photocarcinogenic [72,73].
Sulisobenzone (Benzophenone-4)	UVA and UVB	Linked to oxidative stress and endocrine disruption in fish [66,74,75]. Potential allergen in people [76].

## Data Availability

No new data were created or analysed in this study. Data sharing is not applicable to this article.

## Abbreviations

The following abbreviations are used in this manuscript:

FDA	United States Food & Drug Administration
GRASE	Generally recognised as safe and effective
HSA	Haemangiosarcoma
ROS	Reactive oxygen species
SCC	Squamous cell carcinoma
TiO_2_	Titanium dioxide
UV	Ultraviolet
ZnO	Zinc oxide
